# Feasibility of Diffuse Reflection Spectroscopy for Intraoperative Margin Assessment During Prostatectomy

**DOI:** 10.1016/j.euros.2024.07.112

**Published:** 2024-08-10

**Authors:** Lotte M. de Roode, Lisanne L. de Boer, Marcos Da Silva Guimaraes, Pim J. van Leeuwen, Henk G. van der Poel, Behdad Dashtbozorg, Theo J.M. Ruers

**Affiliations:** a Department of Nanobiophysics, University of Twente, Enschede, The Netherlands; b Image-Guided Surgery, Department of Surgery, Netherlands Cancer Institute-Antoni van Leeuwenhoek Hospital, Amsterdam, The Netherlands; c Molecular Pathology & Biobanking, Netherlands Cancer Institute-Antoni van Leeuwenhoek Hospital, Amsterdam, The Netherlands; d Department of Urology, Netherlands Cancer Institute-Antoni van Leeuwenhoek Hospital, Amsterdam, The Netherlands; e Department of Urology, Amsterdam University Medical Centers, VU University, Amsterdam, The Netherlands

**Keywords:** Diffuse reflectance spectroscopy, Positive surgical margin, Prostate cancer, Robot-assisted laparoscopic prostatectomy, Tissue recognition

## Abstract

**Background and objective:**

A positive surgical margin (PSM) occurs in up to 32% of patients undergoing robot-assisted radical prostatectomy (RARP). Diffuse reflectance spectroscopy (DRS), which measures tissue composition according to its optical properties, can potentially be used for real-time PSM detection during RARP. Our objective was to assess the feasibility of DRS in distinguishing prostate cancer from benign tissue in RARP specimens.

**Methods:**

In a single-center prospective study, DRS measurements were taken ex vivo for RARP specimens from 59 patients with biopsy-proven prostate carcinoma. Discriminating features from the DRS spectra were used to create a machine learning–based classification algorithm. The data were split patient-wise into training (70%) and testing (30%) sets, with ten iterations to ensure algorithm robustness. The average sensitivity, specificity, accuracy, and area under the receiver operating characteristic curve (AUC) from ten classification iterations were calculated.

**Key findings and limitations:**

We collected 542 DRS measurements, of which 53% were tumor and 47% were healthy-tissue measurements. Twenty discriminating features from the DRS spectra were used as the input for a support vector machine model. This model achieved average sensitivity of 89%, specificity of 82%, accuracy of 85%, and AUC of 0.91 for the test set. Limitations include the binary label input for classification.

**Conclusions and clinical implications:**

DRS can potentially discriminate prostate cancer from benign tissue. Before implementing the technique in clinical practice, further research is needed to assess its performance on heterogeneous tissue volumes and measurements from the prostate surface.

**Patient summary:**

We looked at the ability of a technique called diffuse reflectance spectroscopy to guide surgeons in discriminating prostate cancer tissue from benign prostate tissue in real time during prostate cancer surgery. Our study showed promising results in an experimental setting. Future research will focus on bringing this technique to clinical practice.

## Take Home Message

Diffuse reflectance spectroscopy (DRS) is a minimally invasive and nondestructive technology that can be used to determine surgical margin status intraoperatively in real time. Our study results suggest that DRS can potentially be used to detect positive surgical margins during robot-assisted radical prostatectomy.

## 1 Introduction

The primary goal of robot-assisted radical prostatectomy (RARP) is to eradicate prostate cancer while preserving nearby structures and organs involved in sexual function and continence [1]. A positive surgical margin (PSM), defined as the presence of tumor cells on the resected and inked prostate surface [2], occurs in up to 32% of patients undergoing RARP [3]. PSMs are associated with biochemical recurrence that requires additional treatment, and should be prevented [4].

Currently, intraoperative frozen section (IFS) analysis is the only technique routinely available for intraoperative margin assessment [5], [6]. For IFS, areas suspicious for PSM are excised, frozen, and then examined by a pathologist during the prostatectomy procedure. If this examination reveals a PSM, additional tissue is removed at the location of that margin during the same procedure. Although IFS reduces the PSM rate, its clinical use is controversial owing to its low sensitivity (42%) for PSM detection [5], [7]. Neurovascular structure–adjacent frozen section examination (NeuroSAFE) is a specific IFS approach. Instead of a small excision, the entire neurovascular tissue–adjacent circumference is excised and analyzed to ensure complete tumor removal while preserving the neurovascular bundle. NeuroSAFE allows nerve-sparing surgery with high PSM sensitivity (94%), but it is expensive and prolongs the surgery by up to 57 min [8], [9]. In addition, selection of the exact location in the surgical field at risk of PSM is difficult. Thus, there is an unmet need for a technique that allows surgeons to assess surgical margin status intraoperatively with high accuracy and in real time.

Diffuse reflectance spectroscopy (DRS) is an optical technology that can be used to determine margin status intraoperatively in real time. It is based on light reflectance without the need for exogenous contrast agents, so it is minimally invasive and nondestructive [10]. DRS can be used in an in vivo setting without a need for cleaning or staining of tissue. To obtain a DRS measurement, a probe consisting of a source fiber that sends light into the tissue and a detector fiber that collects reflected light from the tissue needs to be brought into contact with the tissue to be measured [10]. DRS can differentiate tissue types on the basis of their unique optical properties. DRS has proven effective in distinguishing tumor tissue from healthy tissue in other fields of cancer research, such as breast cancer (accuracy 95%), colorectal cancer (accuracy 92%), liver cancer (accuracy >90%), lung cancer (accuracy 91%), oral cancer (accuracy 86%), and nerve detection (accuracy 80%) [11], [12], [13], [14], [15], [16], [17].

The aim of our study was to assess the effectiveness of DRS in distinguishing prostate cancer from benign prostate tissue during RARP. We developed an algorithm that classifies tissue types using DRS data collected from freshly excised prostate glands. We then evaluated the performance of this algorithm by comparing its results to those obtained from histological examinations.

## 2 Patients and methods

During this prospective ex vivo study, DRS measurements were taken on cleaved prostate surfaces. The reason for using cleaved prostate glands was to increase the number of tumor measurements in the data set and to create a balanced set of measurements for tumor and healthy tissue. A machine learning algorithm was then developed to classify these measurements on the basis of distinctive features in the DRS spectra.

### 2.1 Patient criteria

This prospective ex vivo feasibility study complied with the Declaration of Helsinki and was approved by the institutional review board (IRBm 19-124) of The Netherlands Cancer Institute-Antoni van Leeuwenhoek Hospital (Amsterdam, The Netherlands). According to Dutch law, no written informed consent from patients was required. We included all patients scheduled for RARP between June 2020 and June 2023 who underwent preoperative staging magnetic resonance imaging (MRI) on which a tumor with a minimum diameter of 10 mm was visible. Patients undergoing androgen deprivation therapy, pelvic radiation therapy, or preoperative indocyanine green injection were excluded. For logistical reasons, patients undergoing NeuroSAFE procedures and those who participated in other clinical trials were excluded. The patients included did not undergo any further selection, so the exact number of patients excluded was not tracked.

### 2.2 DRS set-up

**Fig. 1:**
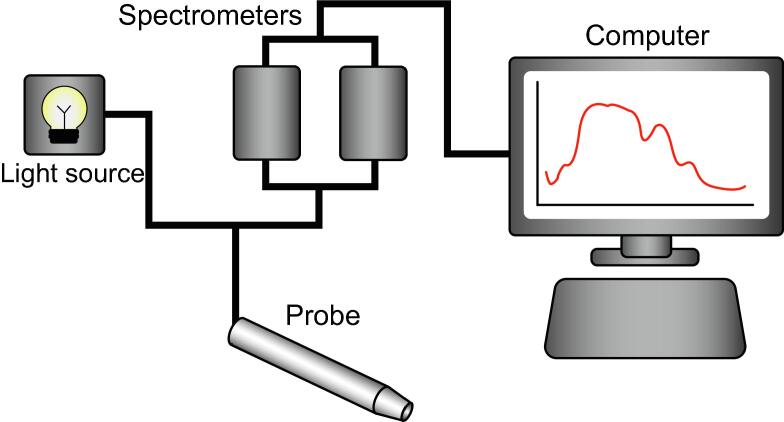
Schematic overview of the set-up. The probe (bottom) is connected to the halogen light source and the spectrometers (visible wavelength range 200–1160 nm, near-infrared wavelength range 900–1750 nm) via two fibers. One fiber functions as an emitting fiber, and the other as a collecting fiber.

The DRS system consisted of the following components: a halogen broadband light source (AvaLight-HAL; Avantes, Apeldoorn, The Netherlands) covering the wavelength range from 360 to 2500 nm; a spectrometer operating in the visible wavelength range from 200 to 1160 nm (AVASPEC-HS2048XL-EVO; Avantes); a spectrometer covering the near-infrared range from 900 to 1750 nm (AVASPECNIR256-1.7-RS; Avantes); and a blunt fiber-optic probe consisting of a source fiber to emit light and a detector fiber to collect light, with a 2 mm source-to-detector fiber distance. This distance was chosen as it results in a measurement volume of up to 2 mm in depth by ∼2 mm in width and ∼2 mm in height and thus provides information on more superficial tissue. The blunt fiber-optic probe was brought into contact with the tissue being measured. Figure 1 provides an overview of the system set-up. The spectra from the two spectrometers were stitched together by the DRS software, resulting in a continuous spectrum from 400 to 1600 nm. Control of the components and data processing were accomplished using custom-built software developed in-house in MATLAB 2023 (MathWorks Inc., Natick, MA, USA). White (Spectralon Avantes WS-2) and dark reference measurements were taken to calibrate the reflectance spectra before data acquisition. Each measurement took approximately 6 s.

### 2.3 Measurement protocol

**Fig. 2:**
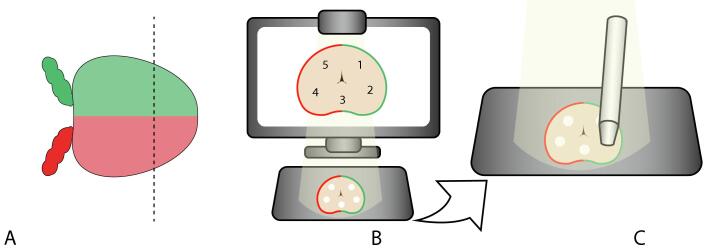
Schematic overview of the measurement protocol. (A) Coronal cut across the prostate. (B) Projection mapping system with selected points on the monitor and their projection on the cut prostate surface. (C) Probe in contact with the prostate tissue to acquire spectra at the selected locations.

Immediately after surgery, whole-mount RARP specimens were collected and inked according to our standard histological protocol (green for the left lobe, red for the right lobe). The prostate specimens were then cut in the coronal plane at the expected tumor location according to preoperative MRI, ensuring that tumor was present on the cut surface of the specimen (Fig. 2A). The prostate specimens were placed under a custom projection mapping system (PMS) that was developed in-house to allow for registration of measurement locations in relation to histopathology results (Fig. 2B) [18]. In brief, the PMS took an image of the specimens with the cut surface facing upwards and then points in the image were manually selected via a separate user interface. The points were chosen on the basis of preoperative MRI results and were spread throughout the prostate to include a variety of locations and tissue types. These points were projected back onto the specimens so that the locations of the projected points could be saved. Depending on the prostate size, five to ten points were projected, and DRS measurements were taken at these points on the cut surface (Fig. 2C).

Following these measurements, the prostate specimens were sent to the histopathology department for standard analysis, which resulted in hematoxylin and eosin (HE) stained images.

### 2.4 Histology correlation and data labeling

**Fig. 3:**
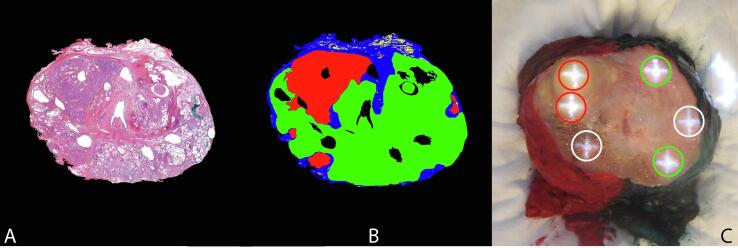
Location of labeling measurements. (A) Digitized and (B) annotated hematoxylin and eosin stained images; tumor is annotated in red, glandular tissue in green, connective tissue in blue, and fat in yellow. (C) Projected measurement locations (crosses). Locations circled in red were labeled as tumor, and locations circled in green as healthy tissue. Locations circled in white were not labeled because of uncertainty regarding tissue type.

The HE slides of measured prostate surfaces were digitized (Fig. 3A), and a pathologist annotated the tissue types: tumor tissue in red, glandular tissue in green, connective tissue in blue, and fatty tissue in yellow. These annotations formed a color-coded label map (Fig. 3B). The annotated HE slides were compared to the snapshot of the cut prostate surface, including the measurement projections. Owing to prostate deformation during histopathological processing, accurate correlation of all measurement locations to the histology was challenging. Therefore, only locations with high certainty were included and labeled as the corresponding tissue type. This included measurement locations in regions with minor deformation and measurement locations in a larger homogeneous area of tissue. Figure 3C shows an example of measurement locations labeled as tumor and healthy tissue in red and green circles, respectively, and uncertain locations, which were excluded, in white circles.

### 2.5 Data analysis

A machine learning model was used for data analysis to achieve discrimination of tumor from healthy tissue on the basis of the DRS spectra acquired. A supervised classification model was trained to predict whether tissue was tumorous or healthy at the measurement locations. The input for the model was based on the spectra acquired. The output of the training model comprises tissue labels predicting tissue type based on the ground-truth pathology results.

Before any analysis, the spectra were normalized using standard normal variate normalization to compensate for variations unrelated to the tissue type [19]. Following normalization, the average combined spectra for tumor tissue and for healthy tissue were calculated.

A feature extraction method was used to prevent overfitting because of the large number of features (1201 wavelengths) in comparison to the number of measurement points. Similar to the approach proposed by de Boer et al [11], average spectra for both tissue types were used to extract features on the basis of local spectral minima/maxima and slopes between wavelength pairs.

A ReliefF feature selection algorithm was applied to the extracted feature set to calculate the feature importance score for all features [20]. ReliefF identifies and ranks important features by comparing them to neighboring data points. Using the ReliefF algorithm, the top-ranked 20 features were selected as the input for the machine learning model.

The data set was split patient-wise into a training set (70%) used to train the model to differentiate between tumor and healthy labels, and a testing set (30%) used to evaluate the final model on data not used for training. The data were split patient-wise to prevent any bias and to ensure that the training and testing sets contained distinct data. A support vector machine (SVM) model with a linear kernel was trained using tenfold cross-validation. This involves dividing the data into ten parts. Nine are used for training and one for validation. Ten model iterations were computed, and the average validation result determines how well the model can generalize to new data. The trained model classified tissue types in the test set, and performance was evaluated using accuracy, sensitivity, specificity, and area under the receiver operating characteristic curve (AUC).

## 3 Results

### 3.1 Data set

A total of 59 patients met the inclusion criteria, leading to 542 individual measurements for healthy and tumor tissues. Of these 542 measurements, 152 could be correlated to the HE slide with a high level of certainty and thus were labeled either as tumor or healthy tissue. The other 390 measurements were discarded. The training set comprised 53 tumor and 47 healthy measurements, while the test set included 27 tumor and 25 healthy measurements. Data set details are summarized in Table 1.

**Table 1:** Overview of data set

Parameter	Result
Labeled measurements, *n* (%)	152 (100)
Tumor	80 (53)
Healthy	72 (47)
Gleason score, *n* (%)	
6	2 (1)
7	123 (81)
8	23 (15)
9	4 (3)

### 3.2 Classification

**Fig. 4:**
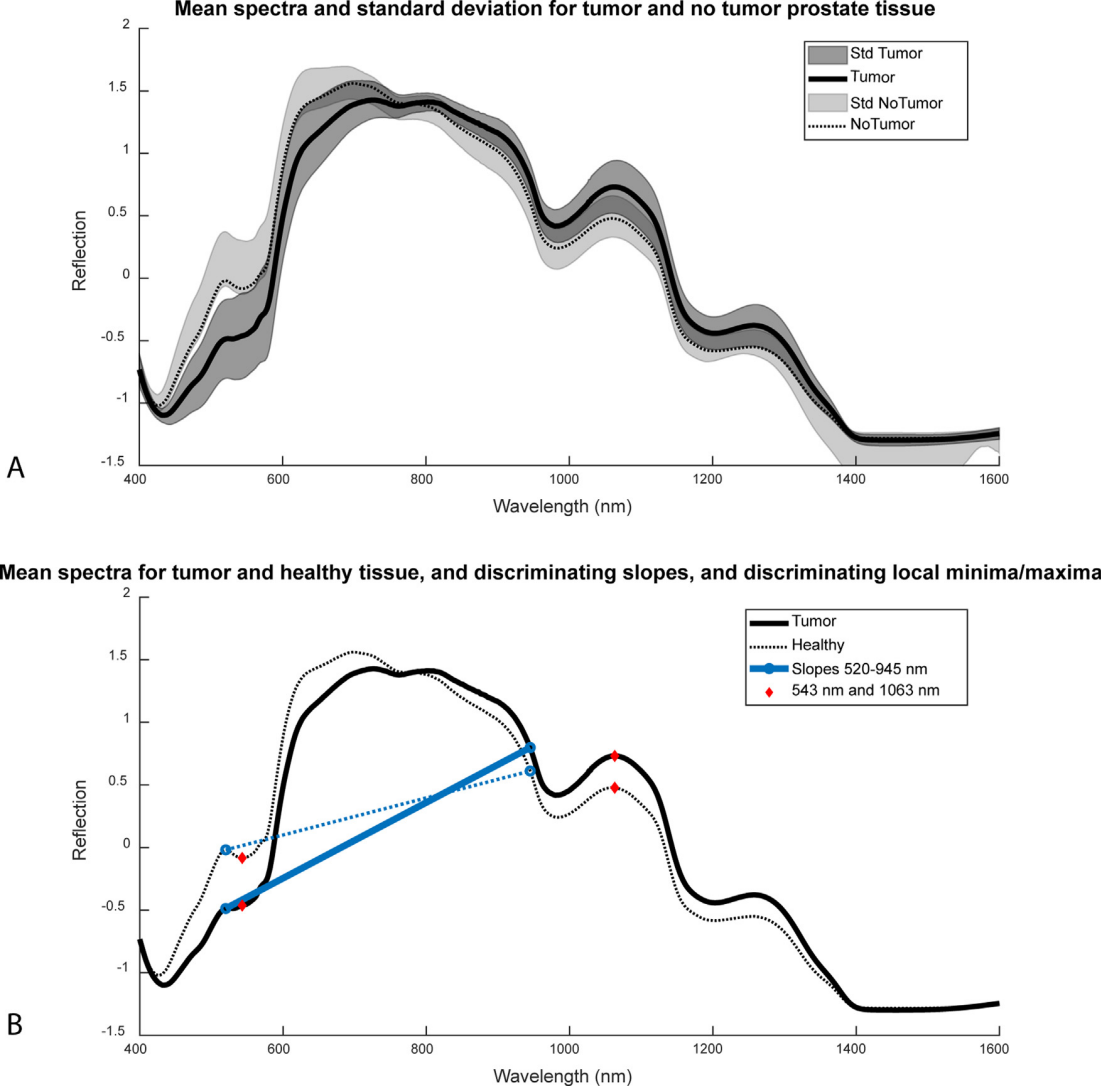
(A) Mean spectra and standard deviation (SD) for tumor locations (dark grey) and healthy locations. (B) Examples of features used as input. Maxima and minima are shown as red diamonds. The continuous (tumor) and dotted (healthy) blue lines show two example slopes for healthy and tumor tissue.

Figure 4A shows average DRS spectra and their standard deviation (SD) for tumor and healthy tissue, with some overlap of SDs. Enhanced discrimination between tumor and healthy tissue is achieved by incorporating multiple slopes and local minima/maxima features, as illustrated in Figure 4B. The ReliefF importance scores for all 20 features are listed in Supplementary Table 1.

**Fig. 5:**
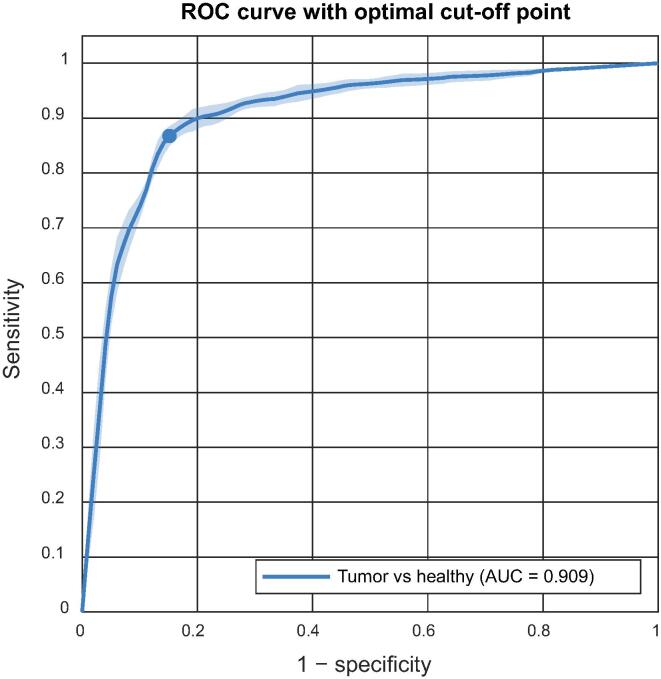
Receiver operating characteristic (ROC) curve with an optimal cutoff point obtained using support vector machine classification for distinguishing tumor tissue from benign prostate tissue. AUC = area under the ROC curve.

After training the model with these features and testing it across ten different data splits, it achieved mean accuracy of 85% (SD 1.0%), mean sensitivity of 89% (SD 0.6%), mean specificity of 82% (SD 1.6%), and mean AUC of 0.91 (SD 0.01), as shown in Figure 5.

## 4 Discussion

We assessed the feasibility of using DRS for tissue discrimination during prostate cancer surgery. Features were extracted from homogeneous tumor and healthy spectra and were used as the input for an SVM classification model. After patient-wise splitting of the data set into a training set and a test set, the model could classify prostate cancer and benign prostate tissue over ten iterations with an average accuracy of 85%. These results suggest that DRS might be capable of detecting PSMs intraoperatively during RARP. Use of DRS could enable surgeons to determine surgical margin status intraoperatively in real time without damaging the tissue measured.

No real-time techniques for PSM assessment are currently available, as NeuroSAFE and IFS require at least part of the specimen to leave the operating theatre for assessment by a trained pathologist [5], [21]. Two other studies reported on DRS use for surgical margin assessment in prostate cancer surgery [22], [23]. These studies showed promising results, but only focused on the visible wavelength range, which is greatly affected by blood perfusion [24]. As a result, the DRS method in these two studies cannot be used in an in vivo setting with blood perfusion, and the assessment can only be conducted after the prostate has been removed, making it very difficult to locate the exact location of a potential PSM on the prostate surface during surgery. We overcame this limitation by using a broad wavelength range (visual and near-infrared) of up to 1600 nm. Thus, our method benefits by focusing on the wavelengths less affected by organ perfusion, so it may be more easy to implement in clinical practice for intraoperative use. Moreover, feature selection means that only selected wavelengths are used to discriminate tissue types. This can potentially facilitate near real-time classification, as the number of wavelengths to be measured and analyzed is limited, which results in faster analysis. The classification algorithm was applied in ten iterations of different training and testing set compositions, contributing to the robustness of the network.

Other optical techniques, such as optical coherence tomography, dual-probe difference specimen imaging, photodynamic diagnosis with 5-aminolevulinic acid, and confocal laser endomicroscopy, have also been proposed for margin assessment [25]. However, these techniques all come with some limitations, such as a need for exogenous contrast agents or rinsing or dying of the specimen, suboptimal diagnostic accuracy, and unsuitability for intraoperative or real-time analysis [26], [27], [28], [29]. By contrast, DRS analysis is possible intraoperatively in real time by using a probe that is compatible with robotic or laparoscopic instruments. In addition, DRS does not require exogenous contrast agents and is thus minimally invasive and nondestructive. One limitation of DRS in comparison to other techniques is the need for tissue contact and the relatively small measurement volume. Therefore, measurement of the entire prostate surface would be time-consuming. However, in continuous measurement configuration, DRS can still be used to measure a larger area within a short time span. Thus, it can be used to aid surgeons in areas for which the possible presence of a PSM is uncertain.

For this feasibility study, we decided to perform measurements on cleaved prostate glands instead of on the uncleaved prostate surface to increase the number of tumor measurements in our data set. However, deformation of prostate tissue may occur during the process to produce HE slides. As a result, we could not label some of our measurement locations, leaving us with 152 measurements on only homogeneous measurement locations (Fig. 3). We could classify these homogeneous measurement volumes with high sensitivity (89%) and specificity (82%). In clinical practice, measurement locations are likely to contain a mixture of glandular, tumor, and connective tissues. Owing to the unbalanced distribution of Gleason grades in the current data set, Gleason grade was not considered in our data analysis. However, it is expected that Gleason grade influences DRS spectra. In addition, when taking measurements from the prostate surface, the prostate capsule and the location of the margin should be considered to obtain a clear understanding of the full applicability of DRS. Therefore, future research should examine the DRS performance in a larger data set of heterogeneous tissue volumes and measurements taken from the prostate surface while considering the measurement site and Gleason grade before the technique can be implemented in clinical practice.

## 5 Conclusions

We investigated the feasibility of using DRS to discriminate prostate cancer from benign prostate tissue during prostatectomy. Our results suggest that DRS can potentially be used to detect PSM intraoperatively during RARP procedures. Before implementing this technique in the clinical setting, future research should focus on using DRS on heterogeneous measurement volumes and on the prostate surface.

  ***Author contributions:*** Lotte M. de Roode had full access to all the data in the study and takes responsibility for the integrity of the data and the accuracy of the data analysis.

  *Study concept and design*: de Roode, de Boer, Ruers.

*Acquisition of data*: de Roode, van Leeuwen, van der Poel.

*Analysis and interpretation of data*: de Roode, de Boer, Da Silva Guimaraes, Dashtbozorg.

*Drafting of the manuscript*: de Roode.

*Critical revision of the manuscript for important intellectual content*: All authors.

*Statistical analysis*: de Roode, Dashtbozorg.

*Obtaining funding*: None.

*Administrative, technical, or material support*: Da Silva Guimaraes.

*Supervision*: de Boer, Ruers.

*Other*: None.

  ***Financial disclosures:*** Lotte M. de Roode certifies that all conflicts of interest, including specific financial interests and relationships and affiliations relevant to the subject matter or materials discussed in the manuscript (eg, employment/affiliation, grants or funding, consultancies, honoraria, stock ownership or options, expert testimony, royalties, or patents filed, received, or pending), are the following: None.

  ***Funding/Support and role of the sponsor:*** None.
