# Supplementary material for: Feasibility of Diffuse Reflection Spectroscopy for Intraoperative Margin Assessment During Prostatectomy
Source: Eur Urol Open Sci. 2024 Aug 10;67:62–8. doi: 10.1016/j.euros.2024.07.112 (PMC11369370; doi:10.1016/j.euros.2024.07.112)
Supplement: Supplementary Table 1 [file mmc1.docx]

**Supplementary Table 1**. Overview of all input features ranked by ReliefF importance score.

| Feature | Importance score |
| --- | --- |
| slope 1111-1115 nm | 0.0538 |
| slope 1155-1158 nm | 0.0374 |
| dip 432 nm | 0.0289 |
| slope 532-536 nm | 0.0281 |
| dip 427 nm | 0.0161 |
| slope 1022-1027 nm | 0.0132 |
| slope 725-734 nm | 0.0116 |
| slope 812-819 nm | 0.0110 |
| dip 1203 nm | 0.0086 |
| dip 762 nm | 0.0076 |
| slope 651-653 nm | 0.0070 |
| dip 769 nm | 0.0069 |
| peak 775 nm | 0.0067 |
| dip 779 nm | 0.0067 |
| slope 611-622 nm | 0.0061 |
| dip 543 nm | 0.0052 |
| peak 1063 nm | 0.0017 |
| slope 520-945 nm | 0.0013 |
| slope 520-1071 nm | 0.0006 |
| peak 524 nm | 0.0005 |
